# An Adaptive Obstacle Avoidance Model for Autonomous Robots Based on Dual-Coupling Grouped Aggregation and Transformer Optimization

**DOI:** 10.3390/s25061839

**Published:** 2025-03-15

**Authors:** Yuhu Tang, Ying Bai, Qiang Chen

**Affiliations:** 1 School of Artificial Intelligence and Big Data, Hefei University, Hefei 230601, China; tangyuhu@stu.hfuu.edu.cn; 2 Anhui JinHai Deer Information Technology Co., Ltd., Hefei 230088, China; 18513451448@163.com

**Keywords:** autonomous robots, grouped aggregation strategy, transformer architecture, Harris hawk optimization, dynamic environment recognition

## Abstract

Accurate obstacle recognition and avoidance are critical for ensuring the safety and operational efficiency of autonomous robots in dynamic and complex environments. Despite significant advances in deep-learning techniques in these areas, their adaptability in dynamic and complex environments remains a challenge. To address these challenges, we propose an improved Transformer-based architecture, GAS-H-Trans. This approach uses a grouped aggregation strategy to improve the robot’s semantic understanding of the environment and enhance the accuracy of its obstacle avoidance strategy. This method employs a Transformer-based dual-coupling grouped aggregation strategy to optimize feature extraction and improve global feature representation, allowing the model to capture both local and long-range dependencies. The Harris hawk optimization (HHO) algorithm is used for hyperparameter tuning, further improving model performance. A key innovation of applying the GAS-H-Trans model to obstacle avoidance tasks is the implementation of a secondary precise image segmentation strategy. By placing observation points near critical obstacles, this strategy refines obstacle recognition, thus improving segmentation accuracy and flexibility in dynamic motion planning. The particle swarm optimization (PSO) algorithm is incorporated to optimize the attractive and repulsive gain coefficients of the artificial potential field (APF) methods. This approach mitigates local minima issues and enhances the global stability of obstacle avoidance. Comprehensive experiments are conducted using multiple publicly available datasets and the Unity3D virtual robot environment. The results show that GAS-H-Trans significantly outperforms existing baseline models in image segmentation tasks, achieving the highest mIoU (85.2%). In virtual environment obstacle avoidance tasks, the GAS-H-Trans + PSO-optimized APF framework achieves an impressive obstacle avoidance success rate of 93.6%. These results demonstrate that the proposed approach provides superior performance in dynamic motion planning, offering a promising solution for real-world autonomous navigation applications.

## 1. Introduction

Autonomous robotics is transforming various domains, including industrial automation, precision agriculture, environmental exploration, and household services. In these scenarios, navigation systems, especially obstacle avoidance capabilities, play a critical role in ensuring successful and safe operations. However, existing obstacle avoidance techniques often lack adaptability in complex or dynamic motion planning. Methods relying on simple sensors and algorithms perform adequately in static environments but exhibit limitations when faced with dynamic motion planning or intricate environmental features.

Recent developments in deep learning (DL) and image segmentation technologies have significantly enhanced the environmental perception capabilities of autonomous robots. Yang [1] proposed an enhanced visual SLAM system named vSLAM-Con, which is specifically designed for dynamic built environments. This system introduces an adaptive dynamic object segmentation method and utilizes an optical flow magnitude-based keyframe selection mechanism to reduce computational overhead while maintaining tracking accuracy. Furthermore, a semantic feature update process was developed to enhance the reliability of tracking features. While these technologies show great potential in fields such as logistics, they typically require large datasets and substantial computational resources. Additionally, achieving real-time obstacle avoidance necessitates high inference efficiency, which remains a significant challenge.

In dynamic built environments, Shabnam et al. [2] integrated deep convolutional neural networks (CNNs) with RTAB-Map to enhance environmental perception. However, this method faces bottlenecks in real-time map updating within dynamic motion planning. Ezugwu A. et al. [3] employed a Siamese CNN for sensorless obstacle avoidance, which improved autonomy but had limited responsiveness to rapid environmental changes. Nejat G. et al. [4] introduced the NavFormer model, an end-to-end deep-learning solution designed for navigation in unknown dynamic motion planning, but it requires large-scale training datasets. Sun, Leyuan, et al. [5] proposed the transfusiondom framework, which combines LiDAR and inertial sensors with a transformer-based fusion approach to improve navigation accuracy while increasing computational complexity. These studies indicate that, although image segmentation techniques have made progress in environmental perception, challenges remain to real-time obstacle avoidance and map updating in dynamic motion planning.

To address these challenges and improve environmental perception and obstacle avoidance in dynamic motion planning [6], this study proposes an innovative algorithm framework named GAS-H-Trans. Building on state-of-the-art methods such as the CAGroup3D framework by Wang et al. [7], the SCAN network by Xu et al. [8], and balanced grouping heads by Zhu et al. [9], we explored the potential of feature grouping and aggregation to enhance model performance. Initially, we developed the GAN-Trans algorithm framework, which leverages a dual-coupling grouped aggregation strategy. This strategy efficiently extracts features through grouped aggregation while concurrently optimizing the Transformer architecture to enhance global feature representation and adaptability to complex scenarios. Subsequently, the Harris hawk optimization (HHO) algorithm was introduced for secondary optimization of the GAN-Trans framework, resulting in the refined GAS-H-Trans algorithm framework, which achieves superior performance in complex environments. Furthermore, the GAS-H-Trans image segmentation framework was integrated with the particle swarm optimization (PSO)-enhanced artificial potential field (APF) method for obstacle avoidance. In the experimental validation of this study, the obstacles remained static throughout the navigation process. Using this method, the autonomous robot dynamically adjusted its obstacle avoidance trajectory based on segmented environmental features. This integration significantly improved the environmental perception capabilities and the accuracy of obstacle avoidance decisions, enabling more efficient navigation in static obstacle environments. The primary contributions of this study include:

(1) **Transformer-based dual-coupling grouped aggregation strategy:**

We proposed the GAS-H-Trans algorithm framework, which integrates a dual-coupling grouped aggregation strategy to optimize feature extraction and the Transformer architecture. By feeding grouped aggregation results into the Transformer and synchronously optimizing its structure, this dual-coupling optimization method significantly enhances feature representation. The proposed framework demonstrates stable performance in complex dynamic motion planning, particularly excelling in detecting large objects and recognizing three-dimensional structures. This method effectively captures both local and long-range dependencies, improving the robot’s overall ability to navigate and avoid obstacles in dynamic motion planning;

(2) **Harris hawk optimization for enhanced transformer framework**

The Harris hawk optimization algorithm was applied to refine the GAS-Trans framework, culminating in the GAS-H-Trans algorithm framework. This approach balances high accuracy with reduced computational resource consumption and improves processing efficiency;

(3) **Intelligent obstacle avoidance in 3D real-world scenarios:**

The GAS-H-Trans framework was applied to intelligent obstacle avoidance tasks in 3D real-world environments. By incorporating the artificial potential field method, the framework enhances obstacle avoidance capabilities in dynamic and complex settings, enabling robots to achieve efficient autonomous navigation in unknown scenarios. To address challenges in traditional artificial potential field methods, we integrate the particle swarm optimization (PSO) algorithm to prevent local minima issues, improving the global stability and performance of the obstacle avoidance system.

This paper is organized as follows. Section 2 reviews related works, focusing on grouped aggregation, Transformer-based optimization, metaheuristic methods, and the integration of image segmentation and potential field methods for dynamic environment obstacle avoidance. Section 3 details the GAS-H-Trans algorithms with a dual-coupling grouped aggregation strategy and the PSO-enhanced artificial potential field method. Section 4 validates the proposed frameworks through experiments, comparing their performances against traditional and state-of-the-art methods. Section 5 summarizes the contributions and discusses potential future research directions. This study aims to provide novel solutions to the challenges of robotic obstacle avoidance, advancing the application of autonomous robotic technologies in complex dynamic motion planning.

## 2. Related Work

(1) **Vision-Based Transformer Applications in Obstacle Avoidance**

Recent advances in deep learning have significantly enhanced the obstacle avoidance capabilities of autonomous robots. Hoshino et al. [10] proposed a CNN-LSTM motion planner for obstacle avoidance in dynamic motion planning. However, this method may experience delays. Zheng et al. [11] introduced a ViT-DPFN-based obstacle detection and MPC-based obstacle avoidance algorithm, providing novel trajectory tracking for underwater autonomous robots, but its performance is limited in dynamic motion planning. To address real-time challenges, Chitta [12] developed a transfusion method, utilizing self-attention mechanisms to integrate image and LiDAR data for better environmental understanding, although computational bottlenecks remain in large datasets. Wang et al. [13] created the NavFormer model to improve navigation in unknown and dynamic motion planning, but the model’s network complexity and parameter tuning still pose challenges. Lin et al. [14] proposed a dual-channel self-attention method (TDCS) to improve obstacle avoidance accuracy and efficiency, although stability issues arise in fast-changing underwater environments. Fan et al. [15] combined ResNet-50 with Transformer modules as a backbone network for semantic segmentation tasks, enhancing model performance in complex environments, especially under varying lighting conditions and rock types. Jing et al. [16] used an ImageNet-pretrained CNN to generate initial coarse masks aimed at extracting foreground regions from input images. Cai et al. [17] applied a transformer-based RMSNet for road scene material segmentation, recognizing various surface materials in road scenes to provide richer visual information for autonomous driving and driver-assistance systems. Zhang et al. [18] proposed the transformer-based visual exploration network (TVENet), enabling mobile robots to effectively explore unknown environments, although further research is needed to enhance its generalization across diverse environments. Therefore, this study focuses on the environmental perception ability of the image segmentation model to improve the accuracy of obstacle avoidance decision-making in autonomous robots.

(2) **Grouped Aggregation Strategy in Robotic Object Detection**

Advances in robotic environmental perception and obstacle avoidance have benefited from innovative feature aggregation methods. Wang et al. [7] introduced the CAGroup3D framework, effectively aggregating fine-grained spatial information via fully sparse convolution RoI pooling, enhancing computational efficiency. Xu et al. [8] proposed the SCAN network, aligning multi-scale sparse features with global voxel-encoded attention, improving regression accuracy for large objects. Zhu et al. [9] developed balanced grouping heads, leveraging sparse 3D convolution for semantic feature extraction and boosting 3D object detection performance. These techniques use feature grouping and aggregation to enhance the detection accuracy of large objects in complex environments while improving computational efficiency. However, they can increase model complexity, requiring more computational resources and resulting in longer training times and more difficult parameter tuning [19,20,21]. Additionally, these methods risk overfitting and may fail to meet real-time requirements in dynamic settings [22,23,24]. In Transformer-based image segmentation tasks, the architecture’s self-attention mechanism helps capture global dependencies, reducing overfitting and enhancing model generalization. Transformers’ strong parallel processing and efficient feature fusion make them suitable for real-time tasks and adaptable to features of varying scales and levels [25,26]. Therefore, this study incorporates feature grouping and aggregation to improve environmental recognition in robotic obstacle avoidance.

(3) **Metaheuristic Optimization for Transformer Frameworks**

Optimization algorithms have shown notable achievements in robotic obstacle avoidance. Hong and Pula [27] combined digital twin technology with particle swarm optimization (PSO) to optimize Shifted Window Transformers. Kumar et al. [28] introduced the GWO-SwinUNet liver segmentation framework using gray wolf optimization (GWO). Toren [29] developed a hybrid gray wolf–whale optimization algorithm, improving dry-type transformer designs for cost reduction and efficiency. Mulla and Gharpure [30] used genetic algorithms (GA) to enhance conversational question-generation frameworks. Although PSO and hybrid methods are easy to implement, they often fall into local optima; GA offers wide applicability but suffers from slow convergence. By contrast, Harris hawk optimization (HHO) delivers higher search efficiency, simpler parameter tuning, and robust performance in solving complex problems. This study applies HHO to optimize the GAN-Trans framework, advancing it into the GAS-H-Trans framework for enhanced dynamic obstacle avoidance.

(4) **The Application of the APF Method in Obstacle Avoidance**

The artificial potential field (APF) method is a classic algorithm widely applied in robotic path planning and obstacle avoidance tasks due to its simplicity and real-time performance. However, traditional APF also faces some limitations, such as susceptibility to local minima and challenges in reaching targets in complex environments. In recent years, various improvements have been proposed to address these issues and enhance the performance and applicability of the APF method. Zhu et al. [31] introduced a new obstacle avoidance method by using slope-based criteria to assess the positional relationship between the robot and obstacles on a plane. This method computes repulsive forces and adjusts their direction to help the robot avoid obstacles. Additionally, by extending this method from a 2D plane to a 3D space using arc interpolation theory, it was optimized for real-world applications by considering the robot’s shape and configuration. Li et al. [32] proposed an improved UAV path-planning algorithm that addresses the unreachable target problem by introducing relative distance into the repulsive force field. This method also adds a corrective force to overcome the local minima and incorporates detection factors to exclude the impact of invalid repulsive forces, thus optimizing the path and shortening its length. Li et al. [33] developed a local obstacle avoidance method based on the improved APF. This method adds virtual target points to avoid large obstacles and establishes droplet-shaped repulsive fields and dynamic obstacle potential fields to enhance the smoothness of the avoidance path. Song et al. [34] proposed an enhanced APF method by adjusting the repulsive force function and introducing relative distance, which resolves the target reachability issue. By combining this with particle swarm optimization (PSO), this approach improves the path-planning efficiency and robustness. Liu et al. [35] designed a distributed robot formation obstacle avoidance system that combines a fuzzy cascade PID controller with the improved APF method. Using LiDAR to detect obstacles, this system ensures stable formation and efficient obstacle avoidance.

Therefore, this study adopts the PSO algorithm to optimize the APF method, effectively avoiding the local minima problem and enhancing the overall stability of obstacle avoidance, ensuring that robots can successfully navigate complex environments and reach their targets.

(5) **Simulation Platforms for Robotic Obstacle Avoidance Validation**

Simulation platforms are integral for developing and validating robotic obstacle avoidance algorithms. Unity3D is ideal for these tasks due to its flexibility, extensibility, realistic simulation environments, efficient integration with ROS/ROS 2, and multi-platform support. Li et al. [36] introduced a method combining mixed reality (MR) technology and deep reinforcement learning (DRL) to generate safe robot motions for human–robot collaboration. Ha and Vinh [37] developed a reinforcement-learning algorithm that integrates Q-learning and deep Q-learning for mobile robot path planning in dynamic motion planning. Their work demonstrated the effectiveness of virtual environments for pre-deployment training and testing. Fernandez-Chaves et al. [38] proposed the Robot@VirtualHome virtual ecosystem, offering a controlled and repeatable testing platform for realistic indoor robot simulations.

Simulation systems built using Unity 2021 LTS [39] and Unity3D-based software [40] provide powerful virtual environments for robotic obstacle avoidance and path planning. These platforms support algorithm development, testing, education, and training, helping users better understand the complexities of robotic obstacle avoidance. Javaid et al. [41] and Diego Guffanti et al. [42] explored digital twin technology’s potential in Industry 4.0. They highlighted its application in robotic obstacle avoidance by creating high-precision virtual environments for strategy simulation and optimization. Zhang et al. [43] proposed a method integrating the ROS robotic operating system with Unity3D software. This enabled interactions between virtual and real robots, offering new possibilities for training and optimizing avoidance algorithms. Additionally, CoppeliaSim [44] is a multifunctional and extensible robotic simulation framework. Integrating actuators, sensors, and controllers, it simplifies deployment complexities and provides efficient testing environments. Aflaki [45] uses reinforcement learning to train a virtual Duckietown agent that can recognize stop signs and stop correctly to improve the safety of autonomous vehicles. Leveraging Unity3D’s versatility and extensibility, this study adopts Unity3D to evaluate the proposed GAS-H-Trans framework’s obstacle avoidance performance in complex and dynamic scenarios.

## 3. Methods

**Figure 1:**
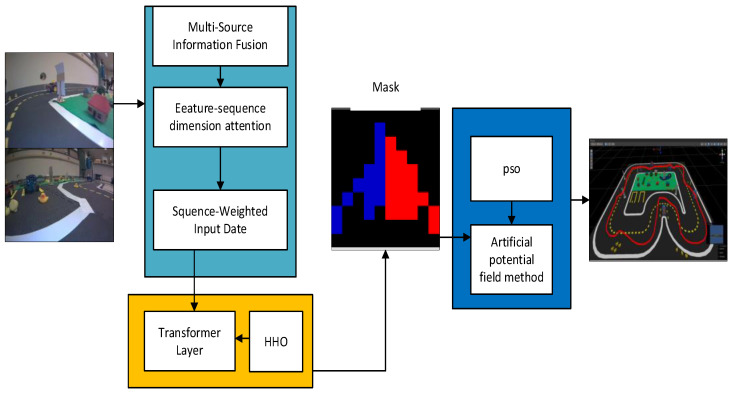
Obstacle avoidance framework based on GAS-H-Trans.

The proposed obstacle avoidance framework is illustrated in Figure 1. This framework leverages the GAS-H-Trans model to enable autonomous obstacle avoidance for robots in simulated environments.

The workflow comprises the following stages:

(1) Data collection:

Industrial-grade cameras are employed to collect image data from simulated environments that replicate real-world scenarios. The collected dataset encompasses diverse environmental variations, including obstacles and intricate layouts, to ensure the robustness of the proposed model. This step aims to provide comprehensive input data for training and evaluation;

(2) Image segmentation using GAS-H-Trans:

The collected images are processed using the GAS-H-Trans framework, which integrates a dual-coupling grouped aggregation strategy to enhance feature extraction. The strategy partitions the input data into subsets, enabling the model to effectively capture fine-grained spatial and semantic details. The Harris hawk optimization algorithm is then applied to refine the Transformer architecture, improving segmentation precision and computational efficiency. The output is a set of detailed segmentation masks, highlighting navigable paths and obstacles;

(3) Obstacle avoidance execution:

The segmentation masks serve as the input to the artificial potential field (APF) algorithms implemented on the Unity3D simulation platform. The APF dynamically computes the robot’s navigation path, enabling it to effectively avoid detected obstacles while maintaining the smoothness of the obstacle avoidance. The PSO algorithm is used to enhance the APF, avoiding local minima issues and improving global path stability. Unity3D simulates realistic obstacle avoidance scenarios for autonomous robots to validate the framework’s adaptability and decision-making capabilities in complex dynamic motion planning.

This comprehensive framework capitalizes on the synergy of dual-coupling grouped aggregation, Transformer optimization, and artificial potential field techniques. The integration ensures obstacle avoidance accuracy for autonomous robots. Simulation experiments conducted on Unity3D validate the robustness of the framework in complex dynamic motion planning.

### 3.1. Overview of GAS-H-Trans Model

The GAS-H-Trans framework integrates a dual-coupling grouped aggregation strategy with Transformer optimization to improve obstacle avoidance in dynamic and complex environments.

**Figure 2:**
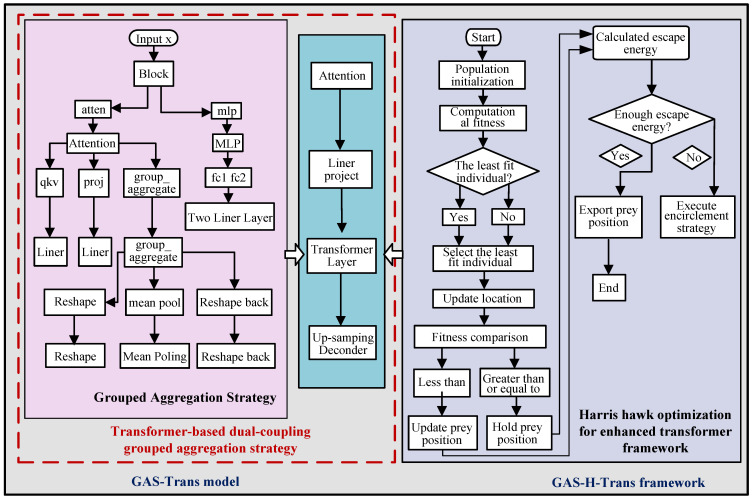
GAS-H-Trans model framework.

As shown in Figure 2, the framework has two main components: GAS-Trans and the enhanced GAS-H-Trans. GAS-Trans introduces grouped aggregation to extract features efficiently and improve Transformer performance. GAS-H-Trans extends this by integrating the Harris hawk optimization (HHO) algorithm to refine the Transformer layers, enhancing image segmentation and obstacle avoidance capabilities.

### 3.2. GAS-Trans Model

The GAS-Trans model leverages a dual-coupling grouped aggregation strategy, as illustrated in Figure 2. Its working mechanism includes the following steps.

**Step 1: Attention Mechanism with Grouped Aggregation**

The combination of multi-head self-attention mechanisms with grouped aggregation achieves a balance between global and local feature extraction. Initially, data pass through a multi-layer perceptron (MLP) for preliminary processing. It then enters the attention module to emphasize critical features. Input sequences are grouped and aggregated to capture fine-grained spatial and semantic information. Two linear layers further refine the extracted features, while reshaping and pooling operations reduce dimensionality and sequence length. This approach enhances computational efficiency.

During grouped aggregation, input sequences X are reshaped and averaged, grouping adjacent patches to reduce sequence length and extract local features. This is expressed as:

(1) $$X_{grouped}=mean(x\;reshaped\;into\;(batch\_size,group\_size,group\_size,\frac{num\_patches}{{group\_size}^{2}},embed\_dim))$$

where X represents the input data, batch_size represents the number of data samples processed simultaneously during each batch, num_patches represent the number of patches, representing the total number of segments into which the input data is divided, embed_dim represents the embedding dimension, which represents the number of features or dimensions for each patch, group_size represent the size of each group, $X_{grouped}$ represents the tensor after grouped aggregation.

For multi-head attention, attention is calculated by combining the query (Q), key (K), and value (V) matrices:

(2) $$MuliHead(Q,K,V)=Concat({head}_{1},{head}_{2},..,{head}_{h})W^{O}$$

For each attention head, the calculation is defined as follows:

(3) $${head}_{i}=Attention(QW_{i}^{Q},KW_{i}^{K},VW_{i}^{V})$$

where Q represents the query matrix, representing the information that needs to be attended to at the current position, K represents the information from all positions. V contains the content or features corresponding to the keys;$\;{head}_{i}$ represents the output of the attention computation for the i head; and $QW_{i}^{Q},\;KW_{i}^{K},\;and\;VW_{i}^{V}$ represent learnable weight matrices used in the computation of Q, K, and V. $W^{O}$ represents a learnable weight matrix used to combine the outputs of multiple attention heads.

For MLP with GELU activation, MLP processes feature within the Transformer block, typically comprising two linear transformations interspersed with a GELU activation function:

(4) $$MLP(X)=GELU(XW_{1}+b_{1})W_{2}+b_{2}$$

(5) Z=LayerNorm(X+Dropout(MultiHead(XQ,XK,XV)))

(6) Z=LayerNorm(Z+Dropout(MLP(Z)))

where X represents the input features. $W_{i}$ and $b_{i}$ denote the learnable weight matrices and bias vectors. GELU represents the Gaussian error linear unit activation function. Z denotes the input features to the Transformer block. MLP(Z) represents the output of the MLP layer. Dropout represents the random dropout operation, and LayerNorm represents layer normalization.

**Step 2: Transformer Layers for Segmentation**

The processed features are input into the Transformer layer for segmentation, leveraging multi-head self-attention to capture dependencies between different regions. Residual connections, layer normalization, and MLP further refine the feature representations.

**Step 3: Output Processing**

The segmentation mask is generated via a classification head and resized to match the input image dimensions. The dual-coupling grouped aggregation strategy ensures robust extraction of both the local and global features, enabling reliable segmentation performance.

### 3.3. HHO-Optimized GAS-H-Trans Model

The HHO-optimized GAS-H-Trans model is an enhancement of the GAS-Trans framework through the integration of the Harris hawk optimization (HHO) algorithm. The HHO algorithm emulates the cooperative hunting behavior of Harris hawks and combines local and global search mechanisms for efficient exploration of the solution space. In this study, the HHO algorithm optimizes key parameters of the Transformer architecture, including the number of layers and iteration steps. The optimization procedure is detailed as follows:

Step 1: parameter initialization: the model’s input and output tensor shapes are defined, and essential parameters such as patch size and embedding dimensions are initialized;

Step 2: Transformer layer optimization via HHO: the parameters for the HHO algorithm, including the number of iterations and population size, are set. The initial population is randomly generated, with each individual representing a potential solution for the Transformer layer configuration. During the iterative process, the fitness of each solution is evaluated based on model performance. The HHO algorithm updates the positions and step sizes of the hawks to converge toward the optimal solution;

Step 3: patch embedding and forward propagation: after determining the optimal Transformer layer configuration, the PatchEmbed module and vision Transformer are constructed. The input images undergo preprocessing and are propagated through the model. The process involves multi-head attention mechanisms, multi-layer perceptron (MLP) operations, and layer normalization. Finally, a classification head generates segmentation masks, which are upsampled if necessary to match the input image size.

By introducing HHO, the computational cost is significantly reduced, and model convergence is accelerated, making it more suitable for real-time applications. The dual-coupling grouped aggregation strategy, combined with the optimized Transformer, delivers superior feature extraction and processing efficiency. The GAS-H-Trans demonstrates exceptional performance in handling complex segmentation tasks, especially in dynamic scenarios.

This optimization pipeline enhances the Transformer structure, significantly improving the image segmentation performance. Algorithm 1 summarizes the optimization procedure.
**Algorithm 1** GAS-H-Trans Algorithm 1. Input: X (a tensor with shape (batch_size, channels, height, width)) 2. Output: Y (the segmentation mask tensor with shape (batch_size, num_classes, height, width)) 3. Start 4. Initialize model parameters: patch_size, embed_dim, num_heads, num_classes, depth (to be optimized), group_size 5. Initialize HHO parameters: hho_iter, hho_pop_size, hho_alpha, hho_beta, hho_gamma 6. Initialize the population of Harris Hawks with random solutions for depth. 7. Evaluate the fitness of each Harris Hawk in the population. 8. While not converged and iteration < hho_iter do   For each Harris Hawk in the population do   Update the Harris Hawk’s position  Update the Harris Hawk’s leader position  Update the step size for each Harris Hawk  Evaluate the new fitness scores for the updated positions  Update the best solution found so far  Select the best depth value for the VisionTransformer model.  Create PatchEmbed module to embed the image patches into a higher dimension.  Initialize the classification token and position encoding. 9. Create the VisionTransformer model with the optimized depth. 10. Preprocess the input image X:  - x = transformer.patch_embed(X) # Embed the image patches  - x = transformer.prepare_tokens(x) # Add classification token and position encoding 11. Forward pass through the VisionTransformer model:  - For each block in the transformer:   - Apply multi-head attention (Equation (2)):    - Compute queries (Q), keys (K), and values (V):     - Q, K, V = self.norm1(x) # Learnable weight matrices as in Equation (3)    - Compute attention outputs for each head:     - For each head i:      - head_i = Attention(Q, K, V) # Equation (3)    - Combine multi-head outputs:     - attention = Concat(head_1, head_2, …, head_h) W^O # Equation (2)   - Apply MLP to further process features (Equation (4)):    - mlp_output = GELU(X W_1 + b_1) W_2 + b_2   - Add residual connections and layer normalization (Equations (5) and (6)):    - residual = x + Dropout(attention)    - z = LayerNorm(residual)    - residual = z + Dropout(mlp_output)    - z = LayerNorm(residual)   - Perform group aggregation with probability group_aggregate_prob (Equation (1)):    - Calculate number of patches per group: per_group = int(sqrt(num_patches/(group_size^2)))    - Reshape x for group aggregation: x_reshaped = x.view(batch_size, group_size, group_size, per_group, per_group, embed_dim)    - Perform aggregation operation: x = mean(x_reshaped) # Equation (1) 12. Apply the classification head to obtain the segmentation mask:   - y = transformer.head(z) 13. If necessary, upsample the segmentation mask to match the original image size:   - y = upsample(y, original_height, original_width) 14. Return the segmentation mask Y 15. End

### 3.4. PSO-Optimized Artificial Potential Field

An optimization strategy for the artificial potential field (APF) method is proposed to solve the potential unreachable target problem in autonomous robot navigation and ensure effective obstacle avoidance. The method redefines the potential field functions to reflect the interactions between the robot, obstacles, and the target more accurately. Unlike traditional methods, this new potential field function considers both the attractive force from the target and the repulsive force from obstacles. The PSO algorithm is used to optimize the attractive and repulsive gain coefficients, allowing the robot to avoid obstacles while moving toward the target.

**Figure 3:**
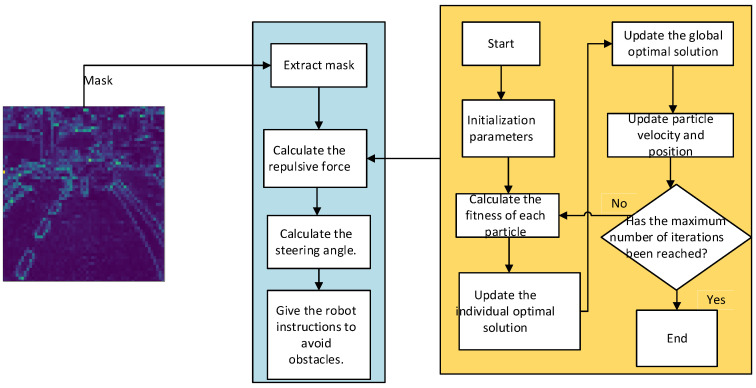
Optimization process of the artificial potential field method.

As shown in Figure 3, the proposed PSO-optimized APF method consists of two main stages. The first stage processes the segmentation mask extracted from the environment. It calculates the repulsive force from the obstacles and determines the steering angles needed for the robot to avoid them. This stage ensures that the robot can move effectively and avoid collisions. The second stage uses the PSO algorithm to optimize the attractive and repulsive gain coefficients. The particles in PSO explore different solutions for these coefficients. The global best solution is updated iteratively, improving the APF’s efficiency and effectiveness. The optimization ensures that the robot maintains smooth motion while avoiding local minima and continuing toward the target.

In detail, the new potential field function can be expressed as:

(7) $$U_{total}=U_{att}+U_{rep}$$

where $U_{att}$ is an attractive potential field and $U_{rep}$ is the repulsive potential field.

The attractive potential field $U_{att}$ is generated by the target point and is mathematically expressed as:

(8) $$U_{att}=\frac{1}{2}k_{att}{\left||p-p_{g}|\right|}^{2}$$

where $k_{att}$ is the attractive gain coefficient, p represents the robot’s current position, and $p_{g}$ is the position of the target. The direction of the attractive force $F_{att}$ points towards the target point, and its magnitude is proportional to the distance between the robot and the target.

(9) $$F_{att}=-\nabla U_{att}=-k_{att}(p-p_{g})$$

The repulsive potential field $U_{rep}$ is generated by obstacles and is expressed as:

(10) $$U_{rep}=\frac{1}{2}k_{rep}{\left(\frac{1}{\rho }-\frac{1}{\rho_{0}}\right)}^{2}$$

where $k_{rep}$ is the repulsive gain coefficient, ρ is the distance from the robot to the obstacle, and $\rho_{0}$ is the influence range of the obstacle. The direction of the repulsive force $F_{rep}$ points towards the robot, and its magnitude is inversely proportional to the distance between the robot and the obstacle:

(11) $$F_{rep}=-\nabla U_{rep}=k_{att}(\frac{1}{\rho }-\frac{1}{\rho_{0}})\frac{p-p_{0}}{\rho^{3}}$$

where $p_{0}$ is the position of the obstacle.

In this study, the PSO algorithm is used to optimize the attractive gain coefficient $k_{att}$ and the repulsive gain coefficient $k_{rep}$ in the artificial potential field (APF) method. By defining a fitness function related to path length, the PSO algorithm searches for the optimal values of $k_{att}$ and $k_{rep}$ to improve the robot’s obstacle avoidance performance. The fitness function can be defined as:

(12) $$F_{\left(k_{att},k_{rep}\right)}=\alpha \cdot L+\beta \cdot T$$

where L is the path length, T is the obstacle avoidance time, and α and β are weight coefficients.

Based on the optimized potential field function, the navigation control is defined. The steering angle is calculated as:

(13) $$\varphi_{t+1}=\varphi_{t}-\gamma \sum_{i,j}{\left(p⊙M_{t}\right)}_{i,j}$$

where $\varphi_{t+1}$ is the steering angle at time step t + 1, $\varphi_{t}$ is the current steering angle at time step t, γ is the weight factor, and P is the symbol mask, with the left half as −1 and the right half as +1. $M_{t}$ is the navigation mask, which contains lane and obstacle information from the segmentation mask, and ⊙ denotes element-wise multiplication.

The pseudocode for the above process is as follows (Algorithm 2):
**Algorithm 2** PSO Optimized Artificial Potential Field for Robot Navigation1. Input:   pred (2D array): Segmentation map with shape (height, width)  robot_position (array): Initial position of the robot [x, y]  target_position (array): Target position [x, y] 2. Output:   trajectory (list of arrays): Path taken by the robot to reach the target 3. Start 4. Initialize parameters:  attractive_weight (float): Weight for the attractive field  repulsive_weight (float): Weight for the repulsive field  repulsive_range (int): Range of the repulsive field  num_particles (int): Number of particles in PSO  num_iterations (int): Number of iterations in PSO  w (float): Inertia weight in PSO  c1 (float): Cognitive (individual) learning factor in PSO  c2 (float): Social (global) learning factor in PSO 5. Define attractive_field(robot_pos, target_pos):  Return attractive_weight * (target_pos robot_pos) 6. Define repulsive_field_pso(robot_pos, obstacle_map, repulsive_range, num_particles, num_iterations, w, c1, c2):  Initialize particles and velocities  Evaluate the initial fitness of particles  For iteration = 1 to num_iterations:   For each particle:    Calculate repulsive force    Evaluate fitness score    Update personal best position and score    Update global best position and score    Update velocities and positions of particles   Return global best position as an optimized repulsive force 7. Define artificial_potential_field(robot_pos, target_pos, obstacle_map, repulsive_range, num_particles, num_iterations, w, c1, c2):   Calculate attractive force   Calculate repulsive force using PSO   Return total force (attractive + repulsive) 8. Define simulate_robot_motion(robot_pos, target_pos, obstacle_map, repulsive_range, num_particles, num_iterations, w, c1, c2, steps):   Initialize trajectory with robot_pos   For step = 1 to steps:    Calculate total force    Update robot position    Append new position to trajectory    If robot reaches target, break   Return trajectory 9. Main program:   Call simulate_robot_motion to get trajectory 10. Visualization:   Plot segmentation map   Plot trajectory   Mark target and start positions 11. End

### 3.5. Evaluation Metrics

(1) Evaluation Metrics for the Performance of the GAS-H-Trans Model

To evaluate the performance of the proposed GAS-H-Trans model, this study employs three key metrics: F1 score, mean intersection over union (mIoU), and accuracy. The formulas for these metrics are provided below:

(14) $$F1=2\times \frac{precision\times recall}{precision+recall}$$

(15) $$IoU=\frac{TP}{TP+FP+FN}$$

(16) $$Accuracy=\frac{Correct\;Predictions}{Total\;Predictions}$$

where precision represents the precision, defined as the ratio of correctly predicted pixels for a specific class (TP) to the total number of pixels predicted as that class (TP + FP); recall represents the recall, defined as the ratio of correctly predicted pixels for a specific class (TP) to the total number of actual pixels in that class (TP + FN); TP (true positives) represents the number of pixels correctly predicted as belonging to the target class; FP (false positives) represents the number of pixels incorrectly predicted as belonging to the target class; FN (false negatives) represents the number of pixels belonging to the target class but missed by the model; correct predictions represents the number of pixels that were classified correctly, and total predictions represents the total number of pixels in the dataset.

(2) Obstacle Avoidance Success Rate

The obstacle avoidance success rate $O_{success}$ is defined as the ratio of the number of successful trials $N_{success}$ in which the robot reaches the target without any collisions to the total number of trials $N_{total}$ conducted. The formula for calculating the obstacle avoidance success rate is given by:

(17) $$O_{success}=\frac{N_{success}}{N_{total}}\times 100\%$$

where $O_{success}$ represents the obstacle avoidance success rate; $N_{success}$ represents the number of successful trials in which the robot successfully avoids obstacles and reaches the target; and $N_{total}$ represents the total number of trials conducted.

This metric provides a direct measure of the robot’s ability to successfully avoid obstacles and reach its destination in various test environments, demonstrating the model’s performance in real-world scenarios.

## 4. Results

### 4.1. Dataset Description

#### 4.1.1. Duckiebot Dataset

The Duckiebot dataset [46] used in this study is a publicly available dataset collected from the Duckietown platform [47]. It comprises 100 RGB images that represent diverse driving scenarios, including lanes, stationary obstacles, and other Duckiebots, making it suitable for autonomous vehicle obstacle avoidance. To enhance diversity, various objects were deliberately placed on the roads during data collection. The dataset is annotated into seven categories: Duckiebot, duckie, white lane markings, yellow lane markings, road signs, and others. The images are divided into 70 training images, 15 validation images, and 15 test images, ensuring sufficient data for training and evaluation.

#### 4.1.2. KITTI Dataset

The KITTI dataset, a collaboration between the Karlsruhe Institute of Technology and the Toyota Technological Institute in Chicago, serves as the benchmark in autonomous driving and computer vision research. Known for its realistic and diverse in-vehicle environment data, it is widely used for evaluating stereo vision, optical flow, visual odometry, 3D object detection, and 3D tracking. In this study, the dataset was split using the train_test_split function into 70% training data and 30% test data. An additional 15% of the training data was set aside for validation. This ensures a final distribution of 70% for training, 15% for validation, and 15% for testing, supporting robust model training and evaluation.

#### 4.1.3. ImageNet Dataset

ImageNet-21k, an extension of the ImageNet project, includes over 140,000 high-resolution images, covering more than 21,000 categories. These categories span common objects to specialized items, providing a wide range of data. The dataset configuration included specifying the dataset path, preprocessing techniques, and batch size in the project setup. The dataset was divided using the train_test_split function into 70% training data, 15% validation data, and 15% test data. This division ensures that the model has ample data for training and enhances the robustness of the model across different scenarios.

To evaluate the effectiveness of the GAS-H-Trans framework, comprehensive experiments were conducted across multiple datasets. These experiments aimed to validate the dual-coupling grouped aggregation strategy and Transformer optimizations.

### 4.2. Performance Comparison of GAS-H-Trans with Baseline Models

This section assesses the overall performance of the proposed GAS-H-Trans framework on Dataset1 by comparing it with baseline models, including CNN, ResNet50, Transformer, and their optimized variants.

We adapted CNN, ResNet50, Mask R-CNN (mask region-based convolutional neural network), and Transformer models for image segmentation by incorporating a segmentation head into their architectures. The CNN and ResNet50 models, which were originally designed for classification, were modified by adding a segmentation head after their backbone networks. This head consists of 1 × 1 convolutions to reduce the feature map’s dimensionality, ReLU activation for non-linearity, and upsampling to recover the segmentation map to the original image resolution. The Transformer model, which processes images through patch embedding and Transformer blocks, also incorporates a segmentation head to convert the feature map into pixel-wise segmentation outputs. Mask R-CNN, unlike CNN and ResNet50, inherently includes a segmentation branch that predicts pixel-wise masks in parallel with object detection. The segmentation branch consists of multiple convolutional layers followed by an upsampling layer to refine the mask predictions, ensuring a precise localization of the segmented regions. The backbone of Mask R-CNN adopts residual connections similar to ResNet50, while its loss function is a combination of cross-entropy loss for classification, smooth L1 loss for bounding-box regression, and average binary cross-entropy loss for mask prediction.

In Table 1, the Conv2d component corresponds to the CNN’s segmentation layers, the shortcut connection refers to ResNet50’s residual connections, and the classification head in the Transformer model is the segmentation head responsible for generating the final pixel-wise segmentation maps. Similarly, in Mask R-CNN, the residual connections contribute to feature extraction, and the segmentation branch directly outputs refined pixel-wise masks.

Additionally, a batch size of 16 was selected, as it provides a good balance between memory efficiency and computational performance. Smaller batches require less GPU memory, making them well-suited for resource-constrained environments while still allowing for parallel processing. Furthermore, batch size 16 contributes to a more stable training process by mitigating the slow convergence and overfitting risks associated with larger batch sizes.

Table 1 presents the model configurations and structural parameters. During model training on Dataset 1, data augmentation techniques were applied to enhance the diversity of the training data and improve the model’s generalization ability. The data augmentation process involves defining and applying a series of image transformations, such as random cropping, translation, rotation, horizontal flipping, and Gaussian blur. These transformations are encapsulated in a custom function and applied to both the images and their corresponding segmentation labels through a custom class method. This approach exposes the model to a wider variety of image variations during training, reducing overfitting and enhancing its adaptability to various changes encountered in real-world scenarios.

Table 2 provides the results for mIoU, F1 score, and accuracy for all models. GAS-H-Trans achieves the best performance across all metrics, as detailed below:

(1) Comparison with CNN and ResNet50:

GAS-H-Trans improves mIoU by 21.8% compared to CNN and 10.5% compared to ResNet50.F1 score increases by 17.6% and 7.5%, respectively. Accuracy improves by 19.5% over CNN and 10.9% over ResNet50. We conducted image segmentation experiments using a CNN-based model on Dataset 1, achieving an mIoU of 63.4%. This represents a 15.7% improvement compared to the 47.7% mIoU reported in Reference [16]. Additionally, the experiment using a ResNet50-based segmentation model on Dataset 1 resulted in an mIoU of 74.7%, which is close to the 78.97% reported in Reference [15]. These results demonstrate that the CNN and ResNet50-based segmentation models used in this study exhibit superior performance;

(2) Comparison with Transformer and optimized variants:

H-Trans1 (HHO-Transformer1), which optimizes Transformer layers, and H-Trans2 (HHO-Transformer2), which optimizes Transformer iterations, show mIoU improvements of 2.5% and 3.3%, and the F1 score increases by 1.8% and 2.3%, respectively. GAS-Trans achieves a 1.2% increase in mIoU and a 0.9% rise in F1 score compared to the original Transformer. GAS-H-Trans surpasses GAS-Trans, with an additional 0.7% improvement in mIoU and a 0.5% gain in F1 score.

GAS-H-Trans achieves the highest mIoU (85.2%), F1 score (91.9%), and accuracy (91.3%) among all of the models. These results highlight the superior accuracy and efficiency of the proposed framework, especially in dynamic and complex environments. Mask R-CNN demonstrates strong instance segmentation capabilities, effectively distinguishing objects. It achieves an mIoU of 75.1% and an F1 score of 84.8%, outperforming conventional CNN and ResNet50 models but still falling short of our proposed GAS-H-Trans model in overall segmentation accuracy. The GAS-H-Trans model has a single-iteration training time of 39 s, while the unoptimized GAS-Trans model takes 35 s per iteration. Although the training time increases with the use of Harris hawk optimization (HHO), the model’s accuracy improves by 4.1%. This improvement significantly reduces the likelihood of errors in the robot’s environmental recognition.

**Figure 4:**
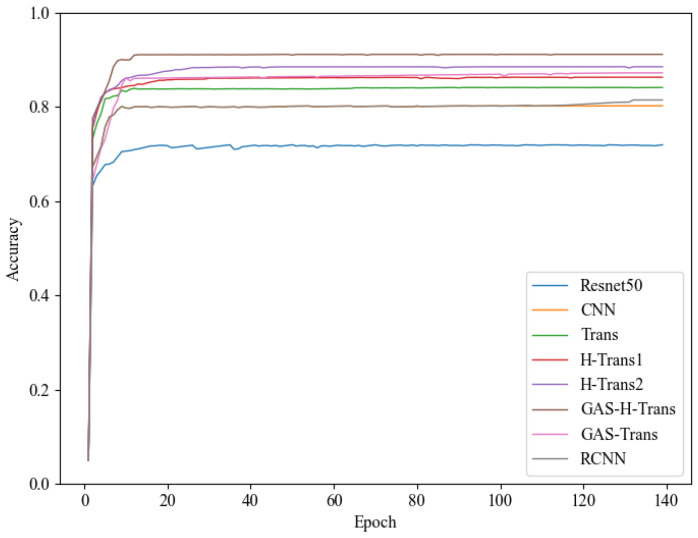
Environment recognition accuracy.

Figure 4 illustrates the accuracy trends of different models over 140 epochs. GAS-H-Trans demonstrates faster convergence and superior accuracy. The results confirm that GAS-H-Trans achieves an optimal balance between accuracy and computational complexity, making it more effective for environment recognition tasks.

**Figure 5:**
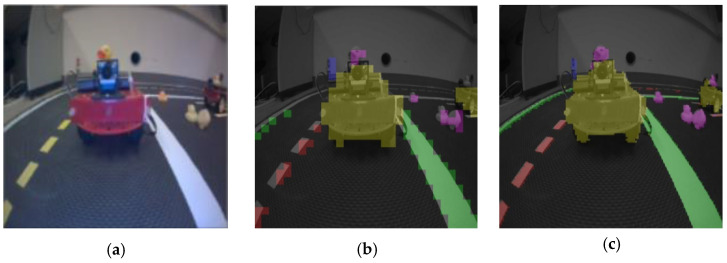
Image segmentation results at 480p resolution. (**a**) The raw input data; (**b**) segmentation result of ResNet50; (**c**) segmentation result of GAS-H-Trans.

Figure 5a presents the raw input image data captured during the experiment. This image is processed using the GAS-H-Trans image segmentation model to refine the environmental features. As shown in Figure 5b,c, the resulting 480P image highlights key objects, such as Duckiebot autonomous robots and obstacles. To further evaluate segmentation performance, we compare GAS-H-Trans with ResNet50 models. Figure 5b illustrates the segmentation output of ResNet50, which, despite its strong feature extraction capability in classification tasks, exhibits limitations in segmenting small objects and complex backgrounds. Figure 5c presents the segmentation result of GAS-H-Trans, which leverages self-supervised pretraining and task-specific optimizations. The results demonstrate that GAS-H-Trans achieves clearer environmental perception with more accurate segmentation, supporting efficient navigation for autonomous robots. These findings highlight the potential of self-supervised learning in reducing reliance on large labeled datasets and underscore the effectiveness of GAS-H-Trans in monocular robotic navigation tasks.

### 4.3. Effectiveness of the Dual-Coupling Grouped Aggregation Strategy

**Figure 6:**
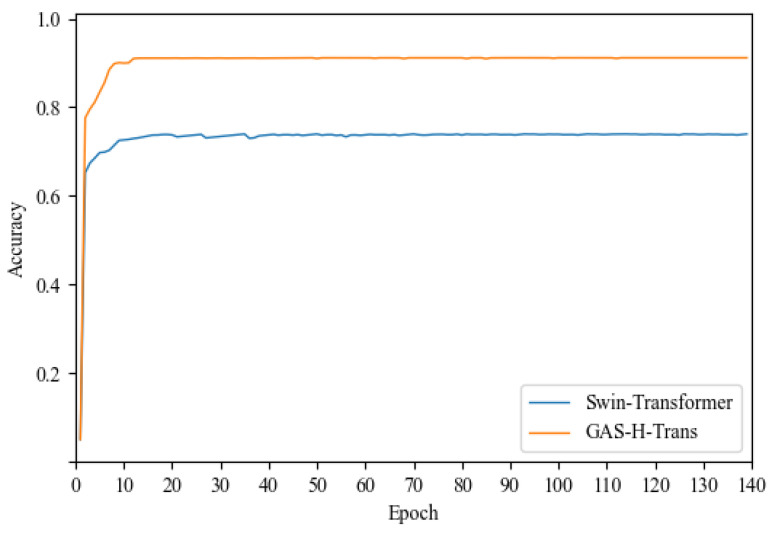
Swin Transformer experiment results.

The GAS-H-Trans framework employs a dual-coupling grouped aggregation strategy, which efficiently extracts features through grouping and optimizes the Transformer structure during aggregation. This approach enhances the global representation capability of the features. In this study, we observed that the Swin Transformer incorporates a window-based multi-head self-attention mechanism (W-MSA) for local feature aggregation and uses a shifted window attention mechanism to achieve global information integration across windows [49]. Both models leverage hierarchical strategies to improve their adaptability to complex environments. This section compares the performance of GAS-H-Trans and the Swin Transformer on Dataset 1, as shown in Figure 6.

Table 3 further illustrates that GAS-H-Trans outperforms Swin Transformer across all three key metrics: mIoU, F1 score, and accuracy. Specifically, GAS-H-Trans improves mIoU by 18.1%, F1 score by 14.7%, and accuracy by 18.1% compared to the Swin Transformer.

The superior performance of GAS-H-Trans is attributed to its innovative grouped aggregation feature extraction mechanism. This mechanism not only effectively captures local features in images but also deeply integrates these features, resulting in more precise global feature representations. The dual optimization of local and global features allows GAS-H-Trans to excel in handling complex scenarios.

In contrast, while the Swin Transformer performs well in processing local features due to its window-based attention mechanism, it is relatively weaker in global information integration. This limitation is particularly evident in complex environments, leading to its lower overall performance compared to GAS-H-Trans.

### 4.4. Generalization Testing of GAS-H-Trans

To evaluate the generalization capability of GAS-H-Trans, its performance was tested on Dataset 2 (ImageNet Real) and Dataset 3 (KITTI). The experimental results demonstrate the robustness and adaptability of GAS-H-Trans across different datasets, highlighting its potential for practical applications. Table 4 presents the mIoU results for GAS-H-Trans compared to CNN/RMSNet algorithms on the two datasets:

The experimental results demonstrate that GAS-H-Trans shows significant performance advantages in image segmentation tasks, especially in the mIoU metric. On the KITTI dataset, GAS-H-Trans achieved an mIoU of 85.2%. This is significantly better than RMSNet (a Transformer-variant model), which achieved an mIoU of 50.34%. The performance improvement is primarily due to the advanced grouped aggregation feature extraction mechanism of GAS-H-Trans. This mechanism effectively captures complex features in urban driving scenarios, enabling more precise obstacle detection in dynamic motion planning. On the ImageNet-21k dataset, GAS-H-Trans also performed excellently, achieving an mIoU of 47.7%. This shows strong robustness and generalization capability. In contrast, traditional Transformer-based methods, while performing well with large-scale and diverse datasets, still fall short of GAS-H-Trans in mIoU. These results further confirm the superiority of GAS-H-Trans in complex environments. Overall, GAS-H-Trans demonstrates significant potential and advantages in real-world autonomous navigation applications.

### 4.5. Effectiveness of GAS-H-Trans in Virtual Robot Obstacle Avoidance

#### 4.5.1. Validation of GAS-H-Trans Model for Image Segmentation in a Virtual Environment

The GAS-H-Trans model was applied to the Unity3D virtual robot environment to assess its performance in image segmentation, simulating real-world obstacle avoidance scenarios. The results confirm the model’s excellent performance in dynamic and complex environments.

**Figure 7:**
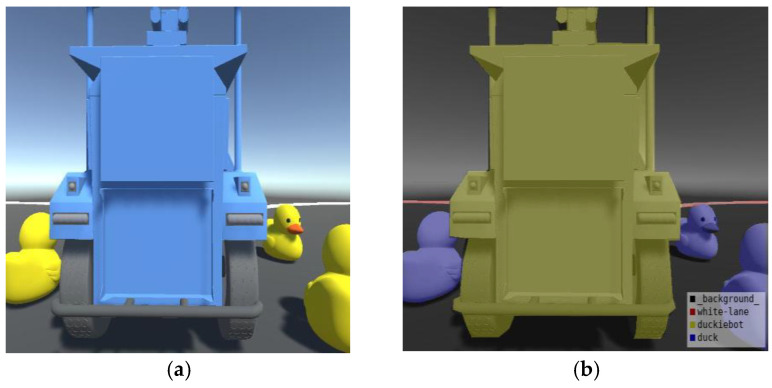
Data construction process. (**a**) Self-built model scene diagram; (**b**) data-labeling chart.

In the experiment, a virtual scene was constructed using 3Dmax and imported into Unity. The scene included a road with 20 randomly placed ducks, 5 road signs, and 5 Duckiebot robots. One Duckiebot was set in motion, while the other objects served as obstacles. Angle data from various positions were recorded and processed. Customized camera scripts were used to capture the image data. Data labeling was performed using LabelMe to complete the dataset. The self-built model scene and data-labeling process are shown in Figure 7.

**Figure 8:**
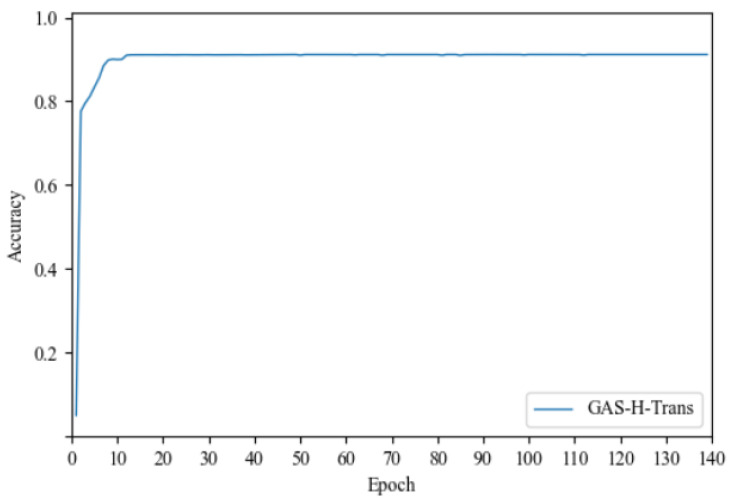
Accuracy of image segmentation on the self-built dataset.

After the dataset was collected, it was used to train the GAS-H-Trans model. The experimental results after training are shown in Figure 8. The accuracy of obstacle feature segmentation reached 89.3% after applying the GAS-H-Trans model to the self-built scene and annotated dataset. This further validates the model’s effectiveness in precise image segmentation for dynamic environmental feature recognition.

#### 4.5.2. Validation of Autonomous Robot Obstacle Avoidance in Virtual Environments with PSO-Optimized APF

(1) Obstacle Avoidance Strategy for Autonomous Robots in Virtual Environments

The segmentation results were input into Unity’s artificial potential field script, enabling the robot to perform obstacle avoidance. The artificial potential field (APF) method is a path-planning algorithm for autonomous robots. It guides the robot to avoid obstacles and reach its goal by simulating attractive and repulsive forces, similar to the gravitational and repulsive forces in the physical world. In this approach, the environment is modeled as a potential field, where the goal location generates an attractive potential, and obstacles generate repulsive potentials. The robot determines its movement direction by calculating the net force’s direction. The APF method is implemented as the controller, and the particle swarm optimization (PSO) algorithm is employed to optimize the attractive and repulsive gain coefficients. This study adopts the PSO algorithm to refine the APF method, effectively avoiding the local minima dilemma and enhancing the overall stability of obstacle avoidance. This ensures that the robot can successfully navigate complex environments and reach its target.

The process of obstacle avoidance is as follows:

(1) Image segmentation: The pre-trained GAS-H-Trans model performs semantic segmentation on images captured by the onboard camera of the robot, rather than being derived from a bird’s-eye view (2D map). It identifies lane markings, obstacles, and other environmental features, providing a structured representation of the environment that improves the accuracy of obstacle recognition and path planning;
(2) Potential field calculation: Based on the segmentation results, the potential field is calculated. The goal position generates an attractive potential, and the obstacles generate a repulsive potential. Unlike traditional sensor-based approaches, this method derives the potential field from vision-based segmentation, ensuring a more detailed and adaptive representation of the environment;
(3) Potential field mapping: The segmentation results are mapped into two potential field masks that represent the left and right sides of the robot, forming a structured spatial representation. These masks correspond to the spatial distribution of virtual forces and are used to simulate the gradient variations of the potential function. They are then transformed into a structured motion-planning coordinate system. Finally, the resultant force synthesized from these potential fields dynamically adjusts the robot’s movement direction, ensuring flexibility and stability in path planning;
(4) Controller design: A potential field-based controller is designed. Using the PSO optimization algorithm to optimize the attractive and repulsive gain coefficients within the APF, the controller calculates a repulsive potential to push the robot away from the side with more obstacles. This controller effectively converts vision-based potential field information into real-world navigation commands, ensuring stability by adapting to dynamic changes in the environment. By incorporating optimization techniques, the controller prevents local minima issues and enhances global path stability;
(5) Obstacle avoidance execution: The robot adjusts its movement based on the controller’s output, successfully performing obstacle avoidance in an autonomous robot operating environment. The control signals, though derived from image-based potential field calculations, are ultimately transformed into robot motion commands in the real-world coordinate system. The robot’s control decisions, including velocity and steering adjustments, are based on the computed potential field forces, enabling precise and adaptive obstacle avoidance.

In contrast to naive APF, which primarily functions as a pure local planner, our method enhances the navigation process by leveraging image-based segmentation information. This allows the robot to make more informed navigation decisions while maintaining the computational efficiency and adaptability of APF-based planning.

In this study, the robot does not use a predefined global map but instead estimates obstacle distances in real-time based on the segmented scene. The specific implementation steps are as follows:

(1) Obstacle identification through segmentation:

The robot identifies obstacles from the segmentation map, categorizing them as regions of high repulsive potential;

(2) Relative positioning within the robot’s field of view:

The detected obstacle’s relative position (x, y) is inferred from its pixel coordinates in the segmentation mask. This position is then converted into the robot’s local coordinate system, allowing APF to compute the required repulsive forces;

(3) APF estimation of distance-based forces:

The APF method indirectly encodes obstacle distance information, where closer obstacles generate stronger repulsive forces and farther obstacles exert weaker repulsive forces. This approach eliminates the reliance on external distance sensors, enabling efficient obstacle avoidance solely through visual data.

**Figure 9:**
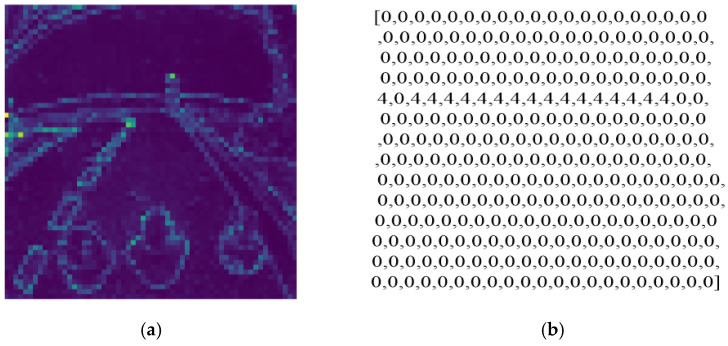
Segmentation mask and its converted array. (**a**) Segmentation mask of GAS-H-Trans; (**b**) partial display of 2D array in JSON Format.

Figure 9 illustrates the segmentation mask generated by the GAS-H-Trans model from images captured by the onboard camera (Figure 9a) and a partial display of the mask data in JSON format (Figure 9b).

In Figure 9a, the different colors correspond to distinct segmented regions or objects, where road surfaces, lane markings, and obstacles are assigned unique color codes. This color-coded segmentation mask enables the robot to accurately recognize key environmental features, facilitating precise navigation and obstacle avoidance.

Figure 9b presents the segmentation data in JSON format, where zero represents free space, while the other numerical values correspond to different objects. This structured data representation provides a clear and interpretable format for real-time decision-making, ensuring that the robot can effectively distinguish between navigable areas and potential hazards in its surrounding environment.

**Figure 10:**
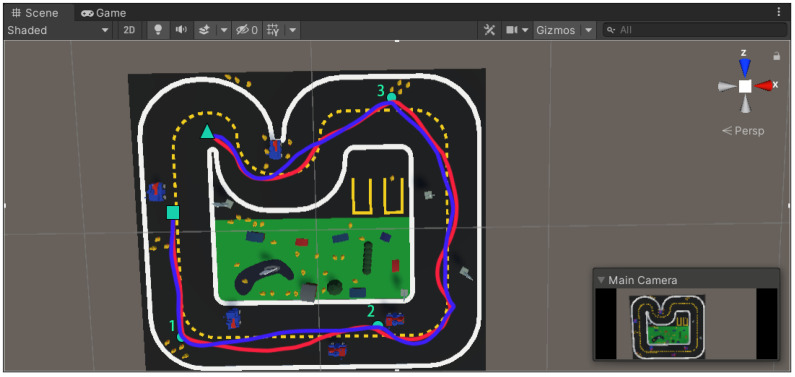
Robot obstacle avoidance in virtual environment.

The final experimental scene, showing the robot’s obstacle avoidance path in Unity, is presented in Figure 10.

An innovative obstacle avoidance strategy is proposed, targeting obstacles that are likely to cause failure in the avoidance process. The strategy involves setting observation points near critical obstacles for secondary precise image segmentation. This two-stage segmentation method significantly enhances the flexibility and accuracy of environmental perception, thus improving the robot’s obstacle avoidance capability in dynamic motion planning.

Based on this innovative strategy, three key reference points, labeled one, two, and three, were set in the experimental scenario, as shown in Figure 10. These points are located near corners and critical obstacles. The green square marks the starting point, and the green triangle marks the goal endpoint. The red curve represents the initial predefined path, while the blue curve shows the robot’s flexible obstacle avoidance path along the predefined route. The yellow dashed line represents the centerline of the road.

From the obstacle avoidance path shown in Figure 10, it is evident that the GAS-H-Trans model can accurately recognize object features, especially in obstacle-dense areas. The blue path is smoother compared to the red path. This demonstrates the stability of the autonomous robot during obstacle avoidance. The smoother path prevents excessive path adjustments or oscillations. This indicates that the PSO-optimized artificial potential field method effectively maintains the robot’s trajectory smoothness and improves obstacle avoidance accuracy. Additionally, Figure 10 illustrates the robot’s adaptability in dynamic motion planning. As the environment changes, the robot can adjust its path flexibly. This helps avoid local minima problems and ensures the stability of the global path. These results confirm the effectiveness of the proposed optimized artificial potential field method and the secondary precise image segmentation strategy, particularly for obstacle avoidance tasks in complex dynamic motion planning.

(2) Obstacle Avoidance Improvement Using the PSO-Optimized APF Method

In this section, we address the potential local obstacle avoidance dilemmas that can occur in dynamic motion planning with traditional artificial potential field (APF) methods. The primary causes of unreachable targets in mobile obstacle avoidance include: (1) when the robot, obstacle, and target are aligned along the same line, the repulsive force from the obstacle and the attractive force from the target counterbalance each other, preventing the robot from moving forward, and (2) the “U-shaped obstacle problem,” where the robot becomes trapped at the bottom of a U-shaped obstacle, and the repulsive forces from both sides of the obstacle are too strong for the attractive force of the target to overcome, preventing the robot from escaping. To resolve these issues, this study proposes a solution that combines the secondary precise image segmentation strategy discussed in the previous section with particle swarm optimization (PSO) to optimize the APF method, thereby enhancing the robot’s ability to navigate complex environments.

In this optimization process, the PSO algorithm is used to fine-tune the attractive and repulsive gain coefficients in the APF. This adjustment allows the robot to avoid the local minima problem and prevents the common issue in traditional APF methods where the robot gets trapped by obstacles.

**Figure 11:**
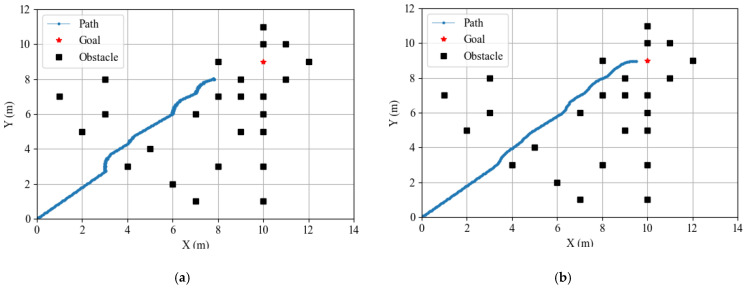
Comparison of obstacle avoidance using the APF method before and after optimization. (**a**) Traditional APF method for obstacle avoidance; (**b**) PSO-optimized APF method for obstacle avoidance.

Figure 11a,b demonstrate the differences in obstacle avoidance performance between the unoptimized and PSO-optimized APF methods. As shown in Figure 11a, the unoptimized APF method causes the robot to stagnate near obstacles and fail to move towards the target, due to the cancellation between the attractive force of the target and the repulsive force of the obstacle. Figure 11b shows the result with the PSO-optimized APF method, where the robot successfully avoids obstacles and reaches the target. The PSO optimization ensures smooth and stable robot movement, resolves the local minima problem, and improves overall navigation efficiency.

**Figure 12:**
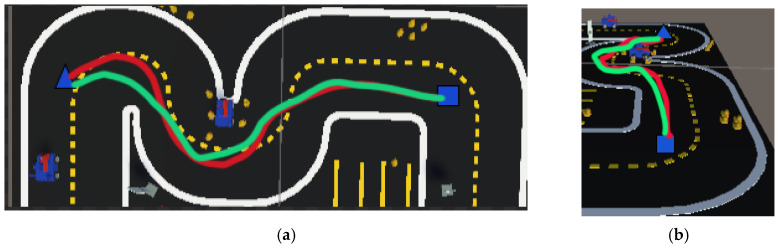
Obstacle avoidance performance with key points. (**a**) Path from top–down view; (**b**) path from side view.

We selected the most challenging obstacle avoidance scenarios from the established virtual environment to perform secondary precise image segmentation at critical points. The PSO-optimized APF method was then applied for autonomous robot obstacle avoidance validation. The results, shown in Figure 12, demonstrate the effectiveness of this approach. The red curve represents the initial predefined path, while the green curve illustrates the optimized obstacle avoidance path. After PSO optimization of the APF parameters, the autonomous robot achieved superior obstacle avoidance performance.

As shown in Figure 12a (top-down view) and Figure 12b (side view), the blue square markers represent observation points, and the blue triangle markers denote the target endpoint. After the PSO optimization, the robot follows the green path, successfully avoiding obstacles and reaching the target efficiently. This validates the effectiveness of the secondary precise image segmentation strategy combined with the PSO-optimized APF method, significantly enhancing the robot’s obstacle avoidance capabilities in complex environments.

#### 4.5.3. Obstacle Avoidance Performance of Autonomous Robots in Virtual Environments

**Figure 13:**
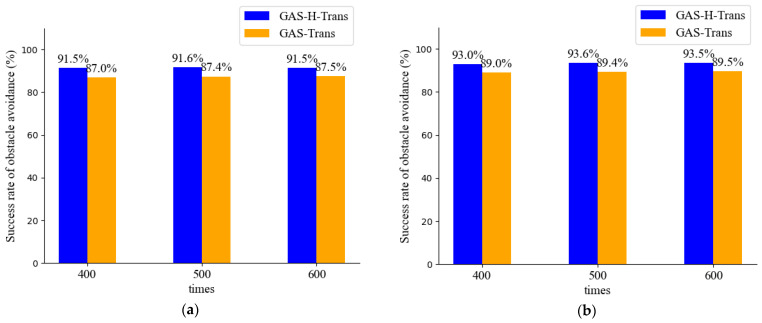
Obstacle avoidance success rate. (**a**) Traditional APF with image segmentation optimization; (**b**) PSO-optimized APF with image segmentation optimization.

We conducted a series of obstacle avoidance experiments in a simulated environment using the full-scene obstacle avoidance mode shown in Figure 10. The obstacle avoidance success rate was used as the evaluation metric. It is defined as the ratio of the number of successful trials in which the robot reaches the target without any collisions to the total number of trials conducted. We recorded the results of 400, 500, and 600 trials, as shown in Figure 13.

Figure 13a demonstrates the obstacle avoidance success rate using the traditional APF method with image segmentation optimization. Figure 13b shows the obstacle avoidance success rate achieved by applying both image segmentation optimization and the PSO-optimized APF method.

The results indicate that the GAS-H-Trans model exhibits outstanding performance. The success rate remained above 91% both before and after the optimization of the APF method, significantly outperforming the GAS-Trans model. After optimizing the APF method, the GAS-H-Trans model’s success rate steadily increased with more test trials, reaching a peak of 93.6%. The GAS-Trans model reached approximately 90%. These obstacle avoidance results effectively validate the efficacy of the proposed strategy, which combines secondary precise image segmentation and the PSO-optimized APF method. It significantly improves the obstacle avoidance performance in complex environments.

## 5. Discussion and Conclusions

In this study, we proposed the GAS-H-Trans framework for image segmentation and dynamic obstacle avoidance in autonomous robots. The key contributions are summarized as follows. **(1) Dual-coupling grouped aggregation strategy:** A Transformer-based dual-coupling grouped aggregation method optimizes feature extraction and enhances global feature representation, thereby improving the model’s perception performance in dynamic motion planning. **(2) Harris hawk optimization (HHO):** The integration of the HHO algorithm into the GAS-Trans framework optimizes the number of Transformer layers and iterations, improving model accuracy and reducing computational costs. **(3) PSO-optimized artificial potential field (APF)**: We integrated the PSO algorithm with APF to optimize the attractive and repulsive gain coefficients, addressing local minima issues and enhancing the global stability of the obstacle avoidance system.

This study also proposes a secondary precise image segmentation strategy. By setting the observation points near critical obstacles for fine-tuned segmentation, the flexibility and accuracy of the segmentation model’s environmental perception are effectively enhanced, thereby improving the robot’s obstacle avoidance capabilities.

Through the integration of PSO-optimized APF with image segmentation, the GAS-H-Trans + PSO-optimized APF framework demonstrated significant improvements in obstacle avoidance. In the experimental validation of this study, the obstacles remained static throughout the navigation process. Using this method, the autonomous robot dynamically adjusted its obstacle avoidance trajectory based on segmented environmental features. This integration significantly enhanced environmental perception capabilities and the accuracy of obstacle avoidance decisions, enabling more efficient navigation in static obstacle environments.

Extensive experiments on publicly available datasets (Duckiebot, KITTI, ImageNet) and in the Unity3D virtual robot environment validate the effectiveness of the proposed framework. The GAS-H-Trans framework outperformed traditional models in image segmentation tasks, achieving the highest mIoU of 85.2%. Furthermore, in virtual obstacle avoidance experiments, the GAS-H-Trans + PSO-optimized APF framework achieved an obstacle avoidance success rate of 93.6%.

These results effectively validate the proposed strategy, which combines secondary image segmentation from GAS-H-Trans with the PSO-optimized APF method, significantly improving obstacle avoidance performance in dynamic motion planning.

Additionally, the GAS-H-Trans framework has the potential to be extended to fully dynamic environments by incorporating real-time object tracking and adaptive obstacle modeling. However, some limitations exist. The majority of the experiments were conducted in simulated environments, and future research will focus on validating the framework in real-world scenarios and improving real-time performance. Additionally, the integration of multi-modal sensor data (such as LiDAR and ultrasonic sensors) will be an important direction for future work to further enhance environmental perception and robustness.

In conclusion, the new framework offers an innovative solution for autonomous robot obstacle avoidance in dynamic motion planning. Its powerful environmental perception and obstacle avoidance performance demonstrate significant potential for practical applications. With further optimization and real-world validation, this framework will play a crucial role in the future development of autonomous navigation and robotics technology.

## Figures and Tables

**Table 1:** Model parameter plot.

Model	Configuration Parameter	Structural Parameters
CNN	Batch size: 16	(Conv2d, BatchNorm2d) × 3 + Conv2d
	Optimizer: SGD	
	Loss Function: Binary Cross-Entropy	Dense
Resent50	Batch size: 50	(Conv2d + BatchNorm2d + Activation) × 3 + Shortcut Connection
	Optimizer: Adam	
	Loss Function: Cross-Entropy Loss	Dense
Mask R-CNN	Batch size: 50	(Conv2d + BatchNorm2d + ReLU) × 3 + Shortcut Connection
	Optimizer: Adam	
	Loss Function: Cross-Entropy Loss+Smooth L1 Loss+Average Binary Cross-Entropy Loss	Dense
Trans	Batch size: 16	Patch Embedding + (Block with Attention + MLP) × Depth + Classification Head
	Optimizer: Adam	
GAS-H-Trans	Batch size: 16	(Linear, BatchNorm) × 3 + Softmax
	Optimizer: Adam	

**Table 2:** F1 value and intersection over union ratio (mIoU) after experimental comparison of different models.

Model	Parameter	mIoU	F1	Accuracy
CNN	1.6 M	63.4%	74.3%	71.8%
CNN [16]	-	47.7%	-	-
Resent50	7.1 M	74.7%	84.4%	80.4%
Resent50 [15]	-	78.97%	-	-
Mask R-CNN		75.1%	84.8%	81.5%
Mask R-CNN [48]	-	60%	68%	74%
Trans	5.8 M	81.2%	89.1%	86.5%
GAS-Trans	5.7 M	82.4%	90%	87.2%
H-trans1	9.8 M	83.7%	90.9%	88.9%
H-trans2	9.8 M	84.5%	91.4%	89.5%
GAS-H-Trans	9.8 M	85.2%	91.9%	91.3%

**Table 3:** Comparison diagram of model parameters.

Model	Parameter	mIoU	F1	Accuracy
GAS-H-Trans	9.8 M	85.2%	91.9%	91.3%
Swin-transformer	9.2 M	67.1%	77.2%	73.2%

**Table 4:** mIoU of ImageNet Real and KITTI datasets running in the GAS-H-Trans model.

Dataset	CNN/RMSNet	GAS-H-Trans
ImageNet Real	47.7% [16]	62.1%
KITTI	50.34% [17]	72.8%

## Data Availability

The original contributions presented in the study are included in the article; further inquiries can be directed to the corresponding author.
